# The association of *EGF* rs2237051 variant, serum EGF levels and generalized aggressive periodontitis: a preliminary study

**DOI:** 10.7717/peerj.9212

**Published:** 2020-05-19

**Authors:** Xian’e Wang, Wenjing Li, Li Xu, Ruifang Lu, Huanxin Meng

**Affiliations:** Department of Periodontology, National Engineering Laboratory for Digital and Material Technology of Stomatology, Beijing Key Laboratory of Digital Stomatology, Peking University School and Hospital of Stomatology, Beijing, China

**Keywords:** Aggressive periodontitis, Epidermal growth factor, Genetics

## Abstract

**Background:**

Epidermal growth factor (EGF) is a pro-inflammatory small peptide that stimulates cell growth, proliferation and differentiation through binding to its receptor. *EGF* rs2237051 and serum EGF levels have been demonstrated to be related with a variety of diseases, including several tumors and inflammatory diseases. Therefore, this study aims to investigate the association of the *EGF* rs2237051 variant and serum EGF levels in Chinese patients with generalized aggressive periodontitis (GAgP).

**Material and Methods:**

A case-control study was conducted among 216 patients with GAgP and 138 healthy controls. The clinical parameters of plaque index, probing depth, attachment loss and bleeding index were recorded. The *EGF rs2237051* polymorphism was genotyped using time-of-flight mass spectrometry, and serum EGF levels were determined. Logistic and linear regression models were used to investigate the association between the genotypes of *EGF* rs2237051, serum EGF levels and GAgP risk.

**Results:**

The AA genotype of *EGF* rs2237051 showed higher risk for GAgP than the combined genotypes GG and AG (adjusted OR = 1.65, 95% CI [1.06–2.57]). Increased serum EGF levels were associated with GAgP (adjusted OR = 1.18, 95% CI [1.14–1.22]). Moreover, the serum EGF level for the AA genotype was significantly higher than that for the AG/GG genotypes in patients with GAgP (adjusted β = 4.70, 95% CI [2.09–7.31]).

**Conclusion:**

We demonstrated that *EGF* rs2237051 variant and the increased level of serum EGF were associated with the risk of GAgP, the serum EGF was up-regulated in patients with GAgP. It was indicated that serum EGF might be a biomarker of GAgP and *EGF* rs2237051 may be related to the genetic background of GAgP.

## Introduction

Generalized aggressive periodontitis (GAgP) is an infectious disease characterized by severe and rapid alveolar bone destruction, which can eventually lead to tooth loss in otherwise healthy, relatively young adults. Although the direct cause for GAgP is bacterial infection, the progression and severity of the disease depend upon the interaction between host genes and environmental factors (Ozer Yucel et al., 2015; Vieira & Albandar, 2014). Lots of studies have demonstrated that genetic factors play an important role in the pathogenesis of aggressive periodontitis (Vieira & Albandar, 2014).

Epidermal growth factor (EGF), a polypeptide molecule, is a multifactorial cytokine that exerts its biological effects, including cell growth, cell proliferation, and wound repair, through binding to cell surface receptors. EGF can stimulate the secretion of collagenase, gelatinase, and plasminogen activators in mucosal keratinocytes. Moreover, EGF can regulate extracellular matrix degradation (Lyons et al., 1993). Severe periodontitis has been shown to be associated with increased activity of collagenase and gelatinase (Birkedal-Hansen, 1993). Chang et al. (1996) demonstrated that EGF-binding capacity in human gingival tissue was enhanced by nearly three-fold during inflammation compared with that in non-inflamed gingiva. Hormia et al. (1993) have demonstrated that the mean EGF concentration in stimulated saliva is slightly higher in juvenile patients with periodontitis than that in healthy controls and proposed that this elevation may be associated with the pathogenesis mechanism of juvenile periodontitis. In addition, it has been reported that the level of serum EGF in patients with rapid progressive periodontitis is significantly higher than that in the healthy controls (Pietruska, Pietruski & Stokowska, 2000). Hence, EGF may be an important mediator in the pathogenesis of periodontitis.

Given the importance of EGF in periodontal diseases, the investigation of genetic polymorphisms that may affect its transcriptional activity can provide important information on its function in periodontal diseases. *EGF* rs2237051 polymorphism is a non-synonymous single-nucleotide polymorphism (SNP) in the coding region of the *EGF* gene that causes a change from isoleucine (ATA) to methionine (ATG) at amino acid position 708. The rs2237051 polymorphism has been demonstrated to be related with several types of cancers, such as lung cancer (Hosgood et al., 2008), advanced esophageal squamous cell carcinoma (Yang et al., 2014) and gastric cancer (Zhan et al., 2013). The rs2237051 polymorphism not only promotes the risk of cancer, but also affects their clinical outcomes. EGF and genetic factors are important for the regulation of the pathogenesis of periodontitis and GAgP, respectively. It is meaningful to investigate the interrelationship between the functional *EGF* rs2237051 polymorphism and GAgP, which maybe further enrich the genetic background of GAgP.

Serum EGF levels have been found to be associated with a variety of diseases, including Parkinson’s disease, several tumors and inflammatory diseases. Considering that periodontal inflammation may cause systemic immune inflammatory response (Cardoso, Reis & Manzanares-Céspedes, 2018), it is necessary to study whether EGF changes in the serum of patients with aggressive periodontitis and the effect of *EGF* rs2237051 polymorphism on the serum EGF concentration.

Therefore, we aimed this study to investigate the association between the *EGF* rs2237051 variant with GAgP, assess the correlation between serum EGF levels and GAgP, and determine the effect of the rs2237051 genotypes on the serum EGF concentration in patients with GAgP.

## Materials and Methods

### Subject population

In the present case-control study, 216 Chinese patients with GAgP and 138 periodontally healthy controls were enrolled. Patients were from the Department of Periodontology at the Peking University School and Hospital of Stomatology and the controls were volunteers from the staff and student population of the hospital. The following clinical and radiographic criteria proposed by the 1999 International World Workshop for a Classification of Periodontal Diseases and Conditions were applied for the diagnosis of GAgP (Armitage, 1999):

- Systematically healthy, except for periodontal disease.
- ≤35 years of age when diagnosed.
- A minimum of eight teeth with probing depth (PD) > 5 mm and attachment loss (AL) > 3 mm and a minimum of threee teeth should not be first molars or incisors among them.

Inclusion criteria for the controls were: ≤35 years of age; PD ≤ 3 mm and on obvious clinical AL; ≤10% of sites with a bleeding index (BI) ≥ 2.

Exclusion criteria for all subjects were: ≥36 years old; smoker; history of periodontal treatment or antimicrobial therapy within 6 months; being pregnant for females; systemic diseases.

This study was approved by the Ethics Committee of Peking University Health Science Center (NO.0313) and was conducted in accordance with the Helsinki Declaration of 1975, as revised in 2013. All subjects had informed written consent questionnaire for the study. Body mass index (BMI) is calculated by weight/height^2^.

### Assessment of clinical parameters

The plaque index (PLI) was scored for buccal and lingual surfaces of all teeth, except the third molars, according to the Quigley & Hein (1962) PLI. PD and AL were measured six sits (mesial, middle and distal sites of the buccal and lingual sites) per tooth except the third molar using Williams periodontal probe. The greatest BI values of the buccal and lingual surfaces were recorded 30 s after probing (Mazza, Newman & Sims, 1981). All the clinical periodontal parameters were recorded by two skilled periodontal specialists (Dong Shi & Li Xu). The calibration was performed on 10 patients with GAgP. The consistency of the replicated measurements of PLI, PD, AL and BI for each examiner (intra-calibration) and paired measurements between the pair of two periodontal specialists (inter-examiner calibration) were recorded. Of the replicated measurements for each examiner, 94.0% (Dong Shi) and 96% (Li Xu) were within 1 unit for PLI; 97.0% (Dong Shi) and 95.8% (Li Xu) were within 1 mm for PD; and 91.5% (Dong Shi) and 93.2% (Li Xu) were within 1 mm for AL, and 97.0% (Dong Shi) and 98.0% (Li Xu) were within 1 unit for BI. Of the paired measurements between the two examiners (Dong Shi vs Li Xu), 92.0% was within 1unit for PLI; 89.8% was within 1 mm for AL, 93.5% was within 1 mm for PD; and 91.6% was within 1 unit for BI.

### Blood collection and assessment of serum EGF levels

A fasted peripheral blood sample was collected and distributed into two tubes from all subjects using venipuncture tubes between 8:00 a.m. and 10:00 a.m. The tube with Ethylenediaminetetraacetic acid (EDTA) was used for white blood cells separation and genomic DNA isolation and the other one without EDTA was applied for measuring serum EGF levels. After 1 h of clotting at 20–26 °C, serum and white blood cells were separated from the blood by centrifugation. The white blood cells and serum were immediately stored at −70 °C until use.

Serum EGF levels were assessed using an enzyme-linked immunosorbent assay kit (R&D Systems, Minneapolis, MN, USA), according to the manufacturer’s instructions. The experiment was performed in duplicate.

### DNA extraction and genotyping

The blood DNA mini kit (Watson Biotechnologies Inc., Shanghai, China) was used for the extraction of genomic DNA from white blood cells, according to the manufacturer’s instructions. The *EGF* rs2237051 single nucleotide polymorphism was genotyped through SEQUENOM MassARRAY matrix-assisted laser desorption ionization time-of-flight (MALDI-TOF) mass spectrometry (Sequenom, San Diego, CA, USA). The genotyping was performed by one investigator who was blind to the periodontal status of all participants.

The primers for the *EGF* rs2237051 genotyping were designed through the Assay Designer software package (Sequenom):

Forward: 5′-ACGTTGGATGGAGGATTATGTGTGGTTCTC-3′

Reverse: 5′-ACGTTGGATGGTACTCTATCTTTGCCAGTC-3′

All genotyping was performed blind to clinical diagnosis by a single investigator.

### Statistical analysis

Continuous variables are presented as mean ± SD, and categorical variables are shown as N (%). The differences between patients with GAgP and healthy controls were assessed using one-way ANOVA (normal distribution) or Mann–Whitney U (skewed distribution) test. Agreement of genotype frequencies with Hardy-Weinberg equilibrium expectations was tested by using a χ^2^ goodness-of-fit test for the controls. Univariate logistic analysis was used to determine the association between the variables, such as age, gender, BMI, genotypes of rs2237051and serum EGF and risk for GAgP. Multiple logistic regression models were used to predict the adjusted odds ratio (OR) at 95% confidence intervals (CI) for the association of the *EGF* rs2237051 variant or serum EGF levels with the risk for GAgP with or without adjusting the covariables. Linear regression models were used to estimate the adjusted coefficient (β) at 95% CI for the association between *EGF* rs2237051 polymorphism and serum EGF levels in healthy controls and patients with GAgP. A two-tailed *P* < 0.05 was considered statistically significant. Statistical analyses were performed using R (http://www.R-project.org) and EmpowerStats software (www.empowerstats.com, X&Y Solutions Inc., Boston, MA, USA).

## Results

The basic characteristics, clinical periodontal parameters, *EGF* rs2237051 genotypes, and serum EGF levels in patients with GAgP and healthy controls are shown in Table 1. The BMI (both continuous and categories) and periodontal parameters (mean PLI, PD, BI and AL) were significantly higher in patients with GAgP than that in healthy controls (*P* < 0.05). The distribution of the *EGF* rs2237051 genotypes was significantly different between the GAgP and control groups. No evidence of deviation from Hardy-Weinberg equilibrium was found for the SNP of rs2237051 (the *P* value of goodness-of-fit test was >0.05). The level of serum EGF was significantly higher in the GAgP group than that in the control group (46.77 ± 9.38 pg/ml vs. 33.54 ± 8.62 pg/ml, respectively, *P* < 0.001). No significant differences were found in age, gender and PLI between both groups.

**Table 1:** Characteristics, genotypes of *EGF* rs2237051 and serum EGF levels in GAgP patients and healthy controls.

Risk of GAgP	GAgP patients	Healthy controls	*P*-value
*N*	216	138	
Age, years	27.29 ± 4.25	27.10 ± 4.23	0.688
Gender			0.859
Female	85 (39.35%)	53 (38.41%)	
Male	131 (60.65%)	85 (61.59%)	
BMI, Kg/m^2^	22.10 ± 3.52	21.28 ± 3.06	0.026*
BMI categories			0.002*
<18.5	26 (12.32%)	20 (14.60%)	
>=18.5, <24.9	138 (65.40%)	106 (77.37%)	
>=24.9	47 (22.27%)	11 (8.03%)	
Mean PLI	2.34 ± 0.39	1.40 ± 0.46	<0.001*
Mean PD, mm	4.82 ± 0.98	1.89 ± 0.61	<0.001*
Mean BI	3.55 ± 0.47	1.17 ± 0.37	<0.001*
Mean AL, mm	4.29 ± 1.44	0.01 ± 0.52	<0.001*
Serum EGF, pg/ml	46.77 ± 9.38	33.54 ± 8.62	<0.001*
Alleles of rs2237051			0.005*
A	311 (71.99%)	171 (61.96%)	
G	121 (28.01%)	105 (38.04%)	
Genotype of rs2237051			0.009*
GG	12 (5.56%)	19 (13.77%)	
AG	97 (44.91%)	67 (48.55%)	
AA	107 (49.54%)	52 (37.68%)	
Genotype of rs2237051			0.029*
AG/GG	109 (50.46%)	86 (62.32%)	
AA	107 (49.54%)	52 (37.68%)	
Genotype of rs2237051			0.008*
GG	12 (5.56%)	19 (13.77%)	
AG/AA	204 (94.44%)	119 (86.23%)	

**Notes:**

* *P* < 0.05, statistical significance.

GAgP, generalized aggressive periodontitis; BMI, body mass index; PLI, plaque index; PD, probing depth; BI, bleeding index; AL, attachment loss; EGF, epidermal growth factor.

Results in table: Mean + SD/N (%).

The univariate analysis data for the risk of GAgP is shown in Table 2. When compared to the individuals with BMI < 18.5 kg/m^2^, individuals with BMI ≥ 24.9 kg/m^2^ has higher odds of periodontitis (OR = 3.29, 95% CI [1.37–7.91]), but no significant difference was found for the individuals with BMI ≥ 18.5 and <24.9 kg/m (*P* > 0.05).

**Table 2:** Univariate analysis for risk of GAgP.

Variables	Statistics	Risk of GAgP	*P*-value
		OR (95% CI)	
Age, years	27.21 ± 4.24	1.01 [0.96–1.06]	0.6875
Gender			
Female	138 (38.98%)	Ref.	
Male	216 (61.02%)	0.96 [0.62–1.49]	0.8587
BMI, Kg/m^2^	21.78 ± 3.37	1.08 [1.01–1.15]	0.0279*
BMI categories			
<18.5	46 (13.22%)	Ref.	
>=18.5, <24.9	244 (70.11%)	1.00 [0.53–1.89]	0.9964
>=24.9	58 (16.67%)	3.29 [1.37–7.91]	0.0079*
Alleles of rs2237051			0.0054*
A	482 (68.08%)	Ref.	
G	226 (31.92%)	0.63 [0.46–0.87]	
Genotype of rs2237051			
GG	31 (8.76%)	Ref.	
AG	164 (46.33%)	2.29 [1.04–5.04]	0.0388*
AA	159 (44.92%)	3.26 [1.47–7.22]	0.0036*
Genotype of rs2237051			
AG/GG	195 (55.08%)	Ref.	
AA	159 (44.92%)	1.62 [1.05–2.51]	0.0292*
Serum EGF, pg/ml	41.61 ± 11.14	1.17 [1.13–1.21]	<0.0001*

**Notes:**

* *P* < 0.05, statistical significance.

Data were presented as OR (95%CI), outcome: Risk of GAgP.

Exposure: Age; BMI; BMI categories; Gender; genotype of rs2237051; serum EGF.

The risk for GAgP was significantly different for the different genotypes of EGF rs2237051. The AG and AA genotypes of EGF rs2237051 increased the risk for GAgP by more than two-fold and three-fold (OR = 2.29; 95% CI [1.04–5.04]; OR = 3.26, 95% CI [1.47–7.22]; respectively) compared to the GG genotype. The AA genotype showed a higher OR (62%) than did the combined genotypes of GG and AG (OR = 1.62; 95% CI [1.05–2.51]). Moreover, serum EGF levels in patients with GAgP were higher than that in healthy controls. Consequently, the risk for GAgP was increased by 17% with one unit increase of serum EGF (OR = 1.17; 95% CI [1.13–1.21]).

The multiple regression analysis data for the genotypes of *EGF* rs2237051 and serum EGF levels for the risk of GAgP are presented in Table 3. The AA genotype showed a higher risk for GAgP than the combined genotypes of GG and AG (adjusted OR = 1.65; 95% CI [1.06–2.57]) after adjusting for age, gender and BMI. Moreover, increased level of serum EGF was found in the patients with GAgP after adjusting for covariables. The risk for GAgP was increased by 18% per unit with an increase in serum EGF levels (adjusted OR = 1.18; 95% CI [1.14–1.22]).

**Table 3:** Multiple regressions of *EGF* rs2237051 and serum EGF for risk of GAgP.

Exposure	Adjust I OR (95% CI)	*P*-value	Adjust II OR (95% CI)	*P*-value
Genotype of rs2237051				
AG/GG (*N* = 195)	Ref.		Ref.	
AA (*N* = 159)	1.64 [1.06–2.54]	0.0263*	1.65 [1.06–2.57]	0.0264*
Serum EGF, pg/ml	1.17 [1.13–1.21]	<0.0001*	1.18 [1.14–1.22]	<0.0001*

**Notes:**

* *P* < 0.05, statistical significance.

Model I was adjusted for age and gender.

Model II was adjusted for age, gender and BMI.

Data were presented as OR (95% CI), outcome: Risk of GAgP.

Exposure: rs2237051; serum EGF.

The association between the genotypes of *EGF* rs2237051 and serum EGF level is shown in Table 4. Among all the participants, individuals with AA genotype of *EGF* rs2237051 had significant higher serum EGF concentration than the subjects with AG/GG genotype (adjusted β = 2.86; 95% CI [0.88–4.84]) after adjustment of age, gender and BMI. While there was no significant difference in the serum EGF concentration among healthy controls with different genotypes of *EGF* rs2237051, among the GAgP patients, individuals with AA genotype of *EGF* rs2237051 had significant higher serum EGF level than the person with AG/GG genotype (adjusted β = 4.70; 95% CI [2.09–7.31]).

**Table 4:** The association between genotype of rs2237051 and serum EGF.

Exposure	Non-adjusted	Model I	Model II
	β (95% CI)	β (95% CI)	β (95% CI)
Total			
Genotype of rs2237051			
AG/GG	Ref.	Ref.	Ref.
AA	3.06 [1.06–5.06]*	2.98 [0.98–4.98]*	2.86 [0.88–4.84]*
Healthy controls			
Genotype of rs2237051			
AG/GG	Ref.	Ref.	Ref.
AA	−0.33 [−3.41 to 2.76]	−0.22 [−3.32 to 2.89]	0.11 [−2.97 to 3.19]
GAgP patients			
Genotype of rs2237051			
AG/GG	Ref.	Ref.	Ref.
AA	5.10 [2.52–7.68]*	4.97 [2.35–7.59]*	4.70 [2.09–7.31]*

**Notes:**

* *P* < 0.05, statistical significance.

Model I was adjusted for age and gender.

Model II was adjusted for age gender and BMI.

Data were presented as β (95% CI), outcome: serum EGF.

Exposure: genotype of rs2237051.

No significant differences were found for the association between *EGF* rs2237051 variants and periodontal parameters, including PLI, BI, PD and AL; and no significant differences were observed for the association between serum EGF levels and periodontal parameters (data not shown).

## Discussion

In the present study, we have demonstrated that there is a significant association between the genotypes of *EGF* rs2237051 and risk for GAgP as well as serum EGF levels, and that serum EGF concentration is increased in patients with GAgP. Our results indicate that the variants of *EGF* rs2237051 are associated with GAgP in Chinese patients. To our knowledge, this is the first study to demonstrate that *EGF* rs2237051 is a susceptibility gene for GAgP. A previous study has reported the association between another intron-specific locus of EGF and severe chronic periodontitis (Suzuki et al., 2004).

The *EGF* rs2237051 (A/G) is a non-synonymous SNP in the coding region of the EGF gene which results in a change from isoleucine (ATA) to methionine (ATG) at amino acid position 708 (I708M). So far, the function of rs2237051 polymorphism has not been well understood and its role in different diseases is not consistent. Zhan et al. (2013) reported that the G allele carriers of rs2237051 is associated with an increased risk of gastric cancer. In another study, the variant carriers of *EGF* rs2237051 was reported to be associated with a markedly decreased risk of lung cancer (Hosgood et al., 2008). In advanced esophageal squamous cell carcinoma patients, the variant allele G of *EGF* rs2237051 was associated with favorable prognosis (Yang et al., 2014). In the present study, we found that the AA genotype of rs2237051 showed the higher serum EGF level and a higher OR (1.62) than did the combined genotypes of GG and AG in Chinese patients with GAgP. The effect of the rs2237051 polymorphism in our study was consisted with other studies (Hosgood et al., 2008; Yang et al., 2014). Amino acid alteration may influence the spatial conformation of the EGF protein and therefore alter the function of the gene. This may explain the correlation between the rs2237051 of the *EGF* gene and GAgP. The possible way via which the polymorphism could actually influence GAgP needs further investigation.

Our study found that serum EGF concentration is increased in patients with GAgP compared with controls. Actually, some studies have reported the association between EGF and periodontitis; however, the results are not consistent in gingival tissues, saliva, gingival crevicular fluid (GCF) and serum. Chang et al. (1996) demonstrated that EGF-binding capacity in human gingival tissue was enhanced by nearly three-fold during inflammation compared with that in non-inflamed gingiva while the EGF level in GCF was significantly lower in the samples collected from pockets >5 mm than in those from pockets <5 mm. Elevated crevicular fluid flow, as a consequence of inflammation, may have a diluting effect on the EGF concentration in GCF. Hormia et al. (1993) have demonstrated that the mean EGF concentration in stimulated saliva is slightly higher in juvenile patients with periodontitis than that in healthy controls. Moosavijazi et al. (2014) demonstrated that the levels of salivary EGF in patients with advanced periodontitis is lower than that in the healthy subjects. However, the sample size of this study was relatively small and only 11 patients with advanced periodontitis, 13 patients with gingivitis and 16 healthy controls were consisted. The technique used to evaluate the results was also different with other studies (Hormia et al., 1993). Another study has shown that the level of serum EGF in patients with rapid progressive periodontitis was significantly higher than that in the healthy controls, which was consistent with our study (Pietruska, Pietruski & Stokowska, 2000). We speculated that the role of serum EGF in patients with GAgP was pro-inflammatory. The possible ways via which EGF could influence periodontitis included that EGF can stimulate IL-1β secretion to induce the production of IL-8 in gingival fibroblasts, which in turn plays an important role in the pathogenesis of periodontitis by increasing the recruitment of immune cells to the inflamed tissue (Yucel-Lindberg & Brunius, 2006). In addition, another study showed that EGF can regulate the expression of MMP-1, -3, -7 and -11 in a dose-dependent manner. This suggests that EGF might play a role in periodontal destruction and wound repair (Cury et al., 2007). In this study, we showed that the level of serum EGF is increased in patients with GAgP, which confirms a positive correlation between serum EGF level and GAgP. The possible reason for the increase of serum levels of EGF in patients with GAgP could be either spillover from the gingival tissues to the peripheral circulation or it could be caused by a systemic inflammatory response to progressive disease in the periodontal pocket.

In our study, we found that when compared to the individuals with BMI < 18.5 kg/m^2^, individuals with BMI ≥ 24.9 kg/m^2^ has higher odds of aggressive periodontitis, which was consist with other studies (Moura-Grec et al., 2014; Ekuni et al., 2008). BMI was also reported to be associated with EGF (Caroleo et al., 2019), so it was regarded as a covariant to analyze the association among the rs2237051 variant, serum EGF and GAgP. However, no significant difference was found after adjusting for BMI. It suggested that the relationship among the rs2237051 variant, serum EGF and GAgP might not be influenced by BMI.

There are some limitations of this study. Firstly, the sample size of this study is limited due to the low incidence of aggressive periodontitis. In addition, according to the study design of epidemiology, the information bias and selection bias might exist in the present study. Besides, when interpreting the results, it must be considered that in order to match the age to GAgP patients, age of the healthy subjects was less than 36 years in the present study. These samples may potentially have been subjects with periodontitis but have not manifested signs and symptoms yet. Indeed, the periodontal healthy subjects should be observed for quite a long time to make sure that they are actually healthy. Furthermore, only one functional SNP of *EGF* was included in this study. Hence, more functional SNPs of *EGF* and the mechanism of how rs2237051 SNP affecting GAgP should be investigated in the future.

## Conclusion

In conclusion, we demonstrated that *EGF* rs2237051 variant and the increased level of serum EGF were associated with the risk of GAgP, the serum EGF was up-regulated in patients with GAgP. It was indicated that serum EGF might be a biomarker of GAgP and *EGF* rs2237051 may be related to the genetic background of GAgP.

## Supplemental Information

10.7717/peerj.9212/supp-1 Supplemental Information 1 Raw data.

## Additional Information and Declarations

### Data Availability

The following information was supplied regarding data availability: The raw measurements are available in a Supplemental File.
